# Epidemiology of virus-induced asthma exacerbations: with special reference to the role of human rhinovirus

**DOI:** 10.3389/fmicb.2014.00226

**Published:** 2014-05-26

**Authors:** Takeshi Saraya, Daisuke Kurai, Haruyuki Ishii, Anri Ito, Yoshiko Sasaki, Shoichi Niwa, Naoko Kiyota, Hiroyuki Tsukagoshi, Kunihisa Kozawa, Hajime Goto, Hajime Takizawa

**Affiliations:** ^1^Department of Respiratory Medicine, School of Medicine, Kyorin UniversityMitaka, Tokyo, Japan; ^2^Gunma Prefectural Institute of Public Health and Environmental SciencesGunma, Japan; ^3^Kumamoto Prefectural Institute of Public Health and Environmental SciencesKumamoto, Japan

**Keywords:** epidemiology, pathophysiology, treatment, human rhinovirus, asthma exacerbation

## Abstract

Viral respiratory infections may be associated with the virus-induced asthma in adults as well as children. Particularly, human rhinovirus is strongly suggested a major candidate for the associations of the virus-induced asthma. Thus, in this review, we reviewed and focused on the epidemiology, pathophysiology, and treatment of virus-induced asthma with special reference on human rhinovirus. Furthermore, we added our preliminary data regarding the clinical and virological findings in the present review.

## INTRODUCTION

More than 200 different types of viruses, such as human rhinovirus (HRV), human metapneumovirus (HMPV), respiratory syncytial virus (RSV), and human parainfluenza virus (HPIV), are known to cause acute respiratory illness (ARI; Tsukagoshi et al., 2013). We recently reported the issue of “virus-induced exacerbation in asthma and chronic obstructive pulmonary disease” (Kurai et al., 2013a), however, among these causative viruses, HRV is now recognized to have a major impact on asthma pathogenesis (Fujitsuka et al., 2011). From this perspective, we reviewed the literature regarding the epidemiology of HRV-induced asthma in adults, together with preliminary epidemiological data obtained at our institution.

## CLINICAL VIROLOGY OF HUMAN RHINOVIRUS

HRV belongs to the genus Enterovirus and family Picornaviridae (Turner and Couch, 2007). HRV possesses a single strand positive-sense RNA (ssRNA) genome of approximately 7.2 kb. The viral capsid is composed of four viral proteins (VP1-4) which are assembled into 60 protomers, resulting in a small icosahedral structure with a diameter of about 28–30 nm (Turner and Couch, 2007). Genetically, HRV is classified into three species; HRV-A, -B, and -C (Simmonds et al., 2010). Furthermore, these species of HRV have more than 150 genotypes (Andries et al., 1990; Arakawa et al., 2012; Kiyota et al., 2013, 2014). Molecular epidemiological studies suggest that the dominant species are HRV-A and -C, while HRV-B is relatively rarely detected (Arakawa et al., 2012; Kiyota et al., 2013). In particular, the VP1 and VP2 proteins have variations in their amino acid sequences, accounting for the large number of viral serotypes (Turner and Couch, 2007). The host receptor for HRV in respiratory epithelial cells is the intracellular adhesion molecule 1 (ICAM-1, CD54) for the 84 major HRV serotypes (HRV-A and -B), or low-density lipoprotein receptor (LDLR) for the other minor HRV serotypes. The receptor for HRV-C is not yet known. It has been suggested that the optimal temperature for replication of HRV is relatively cool (33–35°C), which would limit infections to the upper airway; however, large or medium sized airways lower in the respiratory tract are now also considered cool enough for HRV replication, in spite of the higher temperature of the lung parenchyma (37°C; McFadden et al., 1985). Therefore, HRV is potentially a causative agent of more severe ARI such as bronchiolitis and pneumonia (Turner and Couch, 2007; Watanabe et al., 2010; Smuts et al., 2011; Arakawa et al., 2012), and may be associated with virus-induced asthma (Johnston et al., 1995; Linsuwanon et al., 2009; Fujitsuka et al., 2011; Smuts et al., 2011). HRV might therefore be involved in various ARIs and additional respiratory complications (Kiyota et al., 2013). Lieberman et al. (2009) reported that the detection of any virus include HRV, the sensitivity rates for nasopharyngeal swab (73.3%) was superior than that of oropharyngeal swab (54.2%), respectively.

## VIRUS-INDUCED COLDS AND THEIR NATURAL COURSE AMONG THE GENERAL POPULATION

The common cold is the third most common primary diagnosis in office visits (Hsiao et al., 2010), and this disease is generally self-limiting, usually lasting up to 10 days (Fashner et al., 2012). Among the general population, HRV infection causes common colds at a frequency of 25–53% (Makela et al., 1998; van Gageldonk-Lafeber et al., 2005). Tyrrell et al. (1993) reported that intranasal inoculation with either HRV serotypes 2, 9, and 14, coronavirus type 229E, or RSV in healthy volunteers induced patterns of symptom development which were not substantially different from each other. However, individual signs or symptoms occurred earliest in HRV infections, then in coronavirus, and lastly in RSV, appearing up to 5 days after inoculation, which demonstrated the long incubation periods of RSV in volunteers (Tyrrell et al., 1993).

HRV has been implicated in patients with acute otitis media, exacerbation of chronic obstructive pulmonary disease, common cold, and lower respiratory tract infections in neonates, the elderly and immunocompromised. Arruda et al. (1997) researching the frequency and natural history of HRV infections in adults during autumn, demonstrated that the first symptom noticed most often was sore throat (40%) in HRV culture- or PCR-positive patients, and stuffy nose in HRV-negative patients (27%), using nasal wash specimens. Respiratory symptoms typically develop after 1–2 days after inoculation in studies, and uncomplicated HRV infections usually peak 2–4 days after inoculation. The median duration of HRV colds is 1 week, but up to 25% last more than 2 weeks (Gwaltney et al., 1967; Rotbart and Hayden, 2000). It should be noted that in illness caused by HRV, viral shedding occurs naturally for up to 21 days, but predominantly over a 3–4 days period.

## HRV INFECTION AMONG ASTHMATICS: *IN VIVO* OR *IN VITRO* EXPERIMENTAL STUDIES

HRV-A type16 (HRV-16), a major group virus commonly used for experimental human infection, and HRV-A type1 (HRV-1), which has been used in animal models of HRV infection, are closely related. Grunberg et al. (1999) reported that experimental HRV-16 infection via nasal inhalation leads to a transient decrease of FEV_1.0_ in patients with asthma, and this decreased lung function was correlated with enhanced cold symptoms and / or airway hyperresponsiveness. Contoli et al. (2006) demonstrated that type III interferon (IFN-λ) production levels in *ex vivo* cell cultures derived from bronchial epithelial cells (BECs) and macrophages obtained from asthmatic patients, were lower than in those derived from healthy controls. Furthermore, deficient interferon-λ production was correlated with HRV viral load, severity of clinical symptoms and FEV_1.0_. Message et al. (2008) demonstrated that the severity of intranasally inoculated HRV-induced clinical illness in asthmatic subjects was correlated to virus load and lower airway virus-induced inflammation.

On the other hand, DeMore et al. (2009) reported that no difference in clinical symptoms, and patterns of viral shedding, was noted between subjects with persistent allergic asthma and healthy subjects after experimental infection with HRV. These different results after experimental HRV infection in individual studies in asthmatic patients and healthy subjects might be dependent on the severity of the asthma of those subjects who enrolled in the studies. Indeed, in several reports, neither defective IFN induction by HRV, nor increased HRV replication was observed in primary human BECs derived from subjects with well controlled asthma (Lopez-Souza et al., 2009; Bochkov et al., 2010; Sykes et al., 2014). A few animal models for rhinovirus infection have been showed because a major group of HRV (i.e., HRV-16) did not bind mouse ICAM-1. Only a minor group of HRV (i.e., HRV-1B) infected the mouse. In this regard, Bartlett et al. (2008) generated a transgenic BALB/c mouse expressing a mouse-human ICAM-1 chimeric receptor for HRV-16 infection. This study also showed asthma exacerbation model by intraperitoneally sensitized with ovalbumin with aluminum hydroxide followed by intranasal inoculation of HRV-1B or UV-inactivated HRV-1B.

## HRV AND ASTHMA EXACERBATIONS: CLINICAL FINDINGS

Although data regarding virus respiratory infections (VRIs) as precipitators of asthma attacks in adults are less clear, Nicholson et al. (1993) reported that VRIs are as commonly linked to exacerbations in adults as they are in children (Johnston et al., 1996; Fujitsuka et al., 2011). This study showed that viruses were detected in 44% of clinical exacerbative episodes with a decrease in peak expiratory flow rate (PEFR) of 50 mL/minute or more, and the most commonly identified virus was HRV, followed by coronaviruses and parainfluenza viruses (Nicholson et al., 1993). Thus, the virus most commonly detected in asthma exacerbations appears to be HRV.

Although HRV is well known as the most frequent cause of the common cold, the implications of HRV infection vary according to respiratory diseases. **Table 1** shows the frequency of HRV infection in various adult respiratory diseases such as exacerbation of asthma (Nicholson et al., 1993; Atmar et al., 1998; Tan et al., 2003), common cold (Makela et al., 1998; van Gageldonk-Lafeber et al., 2005), exacerbation of COPD (Seemungal et al., 2001; Rohde et al., 2003; Tan et al., 2003; Beckham et al., 2005; Papi et al., 2006; Hutchinson et al., 2007; Ko et al., 2007; McManus et al., 2008; Kherad et al., 2010; Dimopoulos et al., 2012; Perotin et al., 2013), community acquired pneumonia (Jennings et al., 2008; Johnstone et al., 2008; Johansson et al., 2010; Lieberman et al., 2010; Fry et al., 2011; Wootton et al., 2011; Luchsinger et al., 2013; Takahashi et al., 2013; Huijskens et al., 2014), exacerbation of idiopathic pulmonary fibrosis (Wootton et al., 2011), and asymptomatic infection (Fry et al., 2011).

**Table 1:** HRV infection and its frequency in acute and chronic respiratory diseases in adults.

	Frequency (%)	Reference
Exacerbation of asthma	26–36	Nicholson et al. (1993), Tan et al. (2003), Atmar et al. (1998)
Common cold	25–53	Makela et al. (1998), van Gageldonk-Lafeber et al. (2005)
Exacerbation of COPD	3–27	Tan et al. (2003), Perotin et al. (2013), Rohde et al. (2003), Seemungal et al. (2001), Papi et al. (2006), Hutchinson et al. (2007), Ko et al. (2007), McManus et al. (2008), Kherad et al. (2010), Dimopoulos et al. (2012), Beckham et al. (2005)
Community-acquired pneumonia	2–12	Johnstone et al. (2008), Jennings et al. (2008), Lieberman et al. (2010), Johansson et al. (2010), Takahashi et al. (2013), Luchsinger et al. (2013), Huijskens et al. (2014)
Exacerbation of idiopathic pulmonary fibrosis	5	Wootton et al. (2011)
Asymptomatic infection	2	Fry et al. (2011)

The risk of exacerbations of asthma in adults is elevated after children return to school, and around December 25th (the Christmas holiday in westernized countries), and this is likely to be due to social interactions with children at these times. Prospective monitoring studies using reverse transcription polymerase chain reaction (RT-PCR) indicate that as many as 85% of acute asthma exacerbations in children, and about 60% in adults, were associated with the presence of upper respiratory tract (URT) infections. Corne et al. (2002) found that the detection rates of HRV in asthmatic (10.1%) and healthy participants (8.5%) were similar, but the LRT symptoms were significantly more severe and longer lasting in the asthmatic group than in the healthy group based on one definition of URT and LRT symptoms (**Table 2**; Johnston et al., 1995).

**Table 2:** Respiratory symptoms.

Upper respiratory symtoms	Lower respiratory symptoms
Runny nose	Cough during the day
Sneezing	Cough during the night
Blocked or stuffy nose	Wheeze during the night
Itchy, sore, or watery eyes	Difficulty breathing shortness of breath
Sore throat	
Hoarse voice	
Fever of shivery	
Headaches or face aches	
Aches or pains elsewhere	

*
Cited and adapted from Johnston et al. (1995).*

There is no common antigen across all strains of HRVs; therefore, no reliable diagnostic method for HRV infection has been established using HRV antigens or HRV-specific antibody. Although viral culture is the conventional method for HRV detection, culture methods are not practical in clinical settings for the detection of HRV, because of its slow growing character and requirement for specific culture conditions. Furthermore, the diagnostic capability of molecular amplification techniques such as nucleic acid sequence-based amplification and RT-PCR is superior to those of culture methods (Loens et al., 2006).

## PATHOGENESIS OF HRV-ASSOCIATED ASTHMA EXACERBATIONS

**FIGURE 1:**
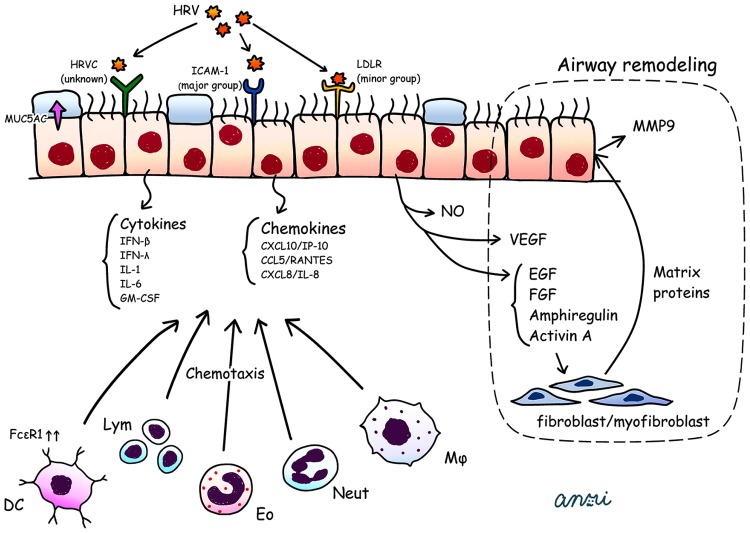
**HRV attaches to airway epithelial cells via ICAM-1, LDLR, and unknown receptors for HRV-C.** HRV infected bronchial epithelial cells secrete a wide range of cytokines (IFN-β, IFN-λ, IL-1, IL-6, GM-CSF) and chemokines (CXCL10/IP-10, CCL5/RANTES, CXCL8/IL-8) together with NO, VEGF, and EGF, FGF, amphiregulin and activin A. These cytokines and chemokines attract various inflammatory cells such as DCs with upregulation of FcεR1, lym, Eo, Neut, and Mϕ. The VEGF, EGF, FGF, amphiregulin and activin A promote the release of matrix proteins from fibroblasts/myofibroblasts, which enhance the production of MMP9 from airway epithelial cells. These phenomena could lead to airway remodeling (dotted area; i.e., thickening of the lamina reticularis). HRV infection induces secretion of MUC5AC, which impairs mucociliary clearance. DCs, dendritic cells; EGF, epidermal growth factor; Eo, eosinophil; FGF, fibroblast growth factor; GM-CSF, granulocyte macrophage colony-stimulating factor; ICAM-1, intercellular adhesion molecule 1; LDRL, low-density lipoprotein receptor; Lym, lymphocyte; MMP, matrix metalloproteinase; Mϕ, macrophage; Neut, neutrophil; NO, nitric oxide synthase; RANTES, regulated on activation, normal T cell expressed and secreted; VEGF, vascular endothelial growth factor.

Experimental HRV infections have been shown to lead to a long-lasting excessive airway narrowing in volunteer subjects with asthma (Cheung et al., 1995; Grunberg et al., 1999). Of note, rhinovirus, unlike influenza and other viruses, causes minimal cytotoxicity (Fraenkel et al., 1995), and the amount of epithelial damage does not correlate with the severity of the symptoms. HRV infection can cause additive or synergistic effects in exacerbation of asthma via the influx of additional inflammatory cells in the airways with preexisted inflammation, resulting in airway cholinergic hyperresponsiveness (Nagarkar et al., 2010), as an allergic response. The effects of HRV infection such as enhanced contractility of airway smooth muscle (ASM) cell and impaired relaxation to cholinergic or β-adrenaergic agonists are attributed solely to binding of the virus to its host receptor ICAM-1 on the ASM cell surface. This proasthmatic-like effect was recognized even in the situation of complete inhibition of viral replication *in vitro*, but not in the setting of pretreatment of ASM with neutralizing antibody directed against for ICAM-1 (Grunstein et al., 2001). Thus, the HRV attachment to ICAM-1 itself can affects the contractility of ASM cells in the absence of any cytopathic effects, and Chun et al. (2013) reported that A 549 cells infected with HRV *in vitro* produced a higher value of IL-8 and RANTES than those of RSV or adenovirus. In addition, only the combination of HRV with Der f1 (house dust mites antigen) acted synergistically to induce IL-8 production. These findings are the reason why the HRV can be a major pathogen for acute exacerbation of asthma. We present a schema for pathogenesis in HRV associated asthma exacerbations (**Figure 1**), which requires the following steps, (1) HRV attachment to airway epithelial cells, (2) an innate immune response which leads to epithelial damage, (3) infection-related airway remodeling.

### ATTACHMENT TO AIRWAY EPITHELIAL CELLS

When RT-PCR is used to either supplement or replace conventional culture techniques, viruses have been found in approximately one half to three quarters of adults experiencing an acute wheezing episode (Jackson and Johnston, 2010), and the majority (59%) of viruses identified were HRVs (Nicholson et al., 1993). However, the evidence is weak, and mechanisms are poorly understood. Initially, HRV-A and -B attach to airway epithelial cells via ICAM-1 or LDLR (Kennedy et al., 2012). The receptor or receptors for the recently identified group HRV-C have yet to be clarified. HRV-infected BECs secrete a wide range of cytokines and chemokines such as IL-1, IL-6, CCL5/RANTES (regulated on activation, normal T cell expressed and secreted), CXCL8/IL-8, GM-CSF, and CXCL10/interferon-inducible protein 10 (IP-10; Jackson and Johnston, 2010; Proud, 2011), which induce neutrophilic, lymphocytic, and eosinophilic inflammation together with airway hyperresponsiveness and airway remodeling (Wark et al., 2002; Proud, 2011).

### THE INNATE IMMUNE RESPONSE

Clearance of viral pathogens begins with interferon secretion, and the underproduction of these factors has been postulated to lead to viral-induced exacerbations. There are three types of interferons, based on the receptors they bind: type I (IFN-α/β), type II (IFN-γ), and type III (IFN-λ). HRV infection induced epithelial expression of mRNA for both type I and type III IFNs, and it has been suggested that impaired epithelial production of IFN-β and IFN-λ in asthmatic subjects may contribute to viral exacerbations of asthma (Wark et al., 2005; Contoli et al., 2006). Contoli et al. (2006) showed significant inverse correlations between *ex vivo* production of IFN-λ and severity of symptoms, bronchoalveolar lavage viral load and airway inflammation, and a strong positive correlation with reductions in lung function during *in vivo* infection. Genome-wide association studies showed that single nucleotide polymorphisms involve in various diseases. Interferon-λ polymorphisms may effect on the incidence of HRV infection (Russell et al., 2014).

Message et al. (2008) reported virus load in asthmatic subjects as being related to increased lower airway inflammation, and in turn increased lower airway inflammation being related to increased symptoms, reductions in lung function, and increases in bronchial hyperreactivity. These data suggest a causal role for HRV infection in the pathogenesis of asthma exacerbations.

Investigating virus-allergen interactions, Durrani et al. (2012) demonstrated that another mechanism that increased expression and cross-linking of the high-affinity IgE receptor, FcεRI, on plasmacytoid dendritic cells is associated with reduced HRV-induced IFN-α and IFN-λ1 secretion, and allergic asthmatic children have significantly reduced HRV-induced IFN-α and IFN-λ1 production after cross-linking of FcεRI.

Type 2, or inducible, nitric oxide synthase (iNOS) is the major NOS isoform found in epithelial cells and can generate substantial amounts of nitric oxide (NO). The NO molecules both inhibit the replication of HRV in airway epithelial cells, and suppresses HRV-induced cytokine production (Proud, 2005). Although the measurement of fractional NO concentration in exhaled breath (FENO) may be used to support the diagnosis of asthma (Dweik et al., 2011), however, increasing of FENO seems to be not always correlated with viral load during the period of HRV infection (Sanders et al., 2004).

Other factors such as allergy, allergen exposure, tobacco smoke, particulates, ozone, stress, and infections such as sinusitis commonly contribute to exacerbations of asthma.

### HRV INFECTION AND AIRWAY REMODELING

Grainge et al. (2011) reported that repeated bronchoconstriction in asthma promotes airway remodeling, and there is now clear evidence that airway remodeling begins in early childhood, and can be present even before clinical diagnosis of asthma is established (Pohunek et al., 2005). Increasing evidence regarding HRV-induced wheezing or exacerbation of asthma raises the possibility that HRV infections could contribute to the initiation and subsequent progression of airway remodeling, which involves multiple factors such as increased epithelial release of Mucin5AC (MU5AC), activin A, amphiregulin, matrix metalloproteinase 9 (MMP9), epidermal growth factor (EGF), fibroblast growth factor (FGF), and vascular endothelial growth factor (VEGF).

HRV infection upregulates production of MUC5AC from epithelial cells, which leads to airflow obstruction in asthma (Hewson et al., 2010). Activin A is a member of the TGF-β superfamily and amphiregulin, a member of the EGF family, alters repair processes (Leigh et al., 2008). Both activin A and amphiregulin have been linked to subepithelial basement membrane thickening in asthma. MMP9 appears to have important roles in asthma exacerbation and airway remodeling (Sampsonas et al., 2007). Expression of VEGF and its receptors is increased in asthmatic subjects, and VEGF is the major proangiogenic activator in asthmatic airways (Feltis et al., 2006; Simcock et al., 2007).

## IMPACTS OF VIRAL INFECTION ON ASTHMA EXACERBATION: PRELIMINARY DATA FROM THE KYORIN COHORT STUDY

Kuga et al. (2000) reported that 61.5% of adult asthmatic patients with common cold suffered an asthma attack, and common cold was significantly associated with acute exacerbations of asthma. They also stated that HRV infection might be important as the virus was detected by RT-PCR in throat gargles (Kuga et al., 2000). Virus-induced exacerbation of asthma is a critical issue for the general physician. However, among asthmatic patients with exacerbative status, distinguishing between those patients which have VRIs, and those who do not, is difficult. Furthermore, epidemiological data regarding adult asthma exacerbations have been sparsely reported. To investigate the prevalence of VRI in exacerbations of adult asthma in both hospitalized or not-hospitalized patients, characterization of clinical and radiological findings was performed. A prospective observational cohort study was conducted at Kyorin University Hospital, Tokyo, Japan from August 2012 to August 2013 (Kurai et al., 2013b). All patients with respiratory symptoms associated with exacerbation of asthma were included, and samples were collected by nasopharyngeal or oropharyngeal swab, and subjected to a PCR method to detect common respiratory viruses. The 44 patients who were enrolled consisted of hospitalized (*n* = 15) or not-hospitalized patients (*n* = 29; **Table 3**). In these two groups, the subject’s backgrounds were similar for age, sex, smoking rates, and duration of illness, however, the measured value of SpO_2_ was significantly lower in hospitalized patients (87 ± 2.3%) than in non-hospitalized patients (96.2 ± 0.7%). The incidence of VRI was significantly higher in the former group (46.7%, *n* = 7) than in the latter group (6.9%, *n* = 2; *p* = 0.002). In the latter group, influenza virus alone was detected in both patients. Furthermore, all hospitalized patients (100%, *n* = 15) had wheezing or severe exacerbation based on the ATS (American Thoracic Society)/ERS (European Respiratory Society) statement (Reddel et al., 2009), whereas, among non-hospitalized patients, only nine patients (31%) were considered as having a severe exacerbation (*p* < 0.001), and 10 patients (38.4%) had wheezing (*p* < 0.001). These findings suggested that virus infection was certainly associated with the hypoxemia and / or wheezing which resulted in a severe or serious asthma attack, based on the Japanese guidelines (Ohta et al., 2011) or the ATS/ERS statement (Reddel et al., 2009). Previous studies using PCR-based viral diagnostics found that viral respiratory infections were detected in up to 85% of exacerbations of asthma in children and about 50% of exacerbations in adults (Nicholson et al., 1993; Johnston et al., 1995), which is similar to our results. Serum inflammatory or allergic markers are not different between the hospitalized and non-hospitalized patients (**Table 3**).

**Table 3:** Comparison of the clinical characteristics of hospitalized and non-hospitalized asthma attack patients.

	Hospitalized patients	Non-hospitalized patients	
Number of cases	15	29	Total 44
Number of virus positive cases	46.7% (*n* = 7)	6.9% (*n* = 2)	*p* = 0.002^**^
Age	52 ± 5.8	60 ± 3.2	NS
Sex (M/F)	5/10	10/19	NS
Smoker	33.3% (*n* = 5)	24.1% (*n* = 7)	NS
Duration of illness (years)	20.6 ± 4.7	18.0 ± 4.4	NS
SpO_2_(%)	87.0 ± .2.3	96.2 ± 0.7	*p* < 0.001^***^
Wheezing	100% (*n* = 15)	38.4% (*n* = 10)	*p* < 0.001^***^
Severe or serious asthma attack on Japanese guideline^†^	80% (*n* = 12)	6.9% (*n* = 2)	*p* < 0.001^***^
Severe attack on ATS/ERS statement^††^	100% (*n* = 15)	31% (*n* = 9)	*p* < 0.001^***^
WBC(/μl)	10,028 ± 1,568	9,850 ± 2,220	NS
CRP(mg/dL)	4.1 ± 1.5	1.1 ± 0.15	NS
lgE(IU/mL)	687 ± 191	545 ± 191	NS

† Defined by Ohta et al. (2011)

†† defined by Reddel et al. (2009).

** *p* < 0.01

*** *p* < 0.001. All data are presented as (mean ± SD).

In hospitalized patients, the viruses identified were HRV (*n* = 5), HMPV (*n* = 1), and RSV (*n* = 1). At the time of admission, the virus-positive group (*n* = 7) had significant lower values of SpO_2_ (81.4 ± 3.9%) than those of the virus-negative group (*n* = 8, SpO_2:_ 91.8 ± 1.3%, *p* < 0.007), and for the patients whose data are available, the frequency of hypercapnea (PaCO_2_ ≧ 45 Torr) was significantly higher in the virus positive group (66.7%, *n* = 4) than in the virus negative group (0%; *p* = 0.014; **Table 4**). The mechanisms for hypercapnea in virus infected individuals have not been elucidated. However, Cheung et al. (1995) reported that HRV infection causes long lasting excessive airway narrowing in response to methacholine in asthmatic subjects. We speculated that smooth muscle might have a role in exaggerated airway narrowing in virus positive asthmatic patients, as described by King et al. (1999).

**Table 4:** Clinical characterization of hospitalized patients with asthma attack based on the presence of virus infection.

	Virus positive	Virus negative	
Number of hospitalized patients	7	8	Total 15
Age	49.4 ± 8.8	54.3 ± 8.2	NS
Sex (M/F)	3/4	3/6	NS
Smoker	28.6% (*n* = 2)	37.5% (*n* = 3)	NS
Duration of illness (years)	28 ± 5.8	11.4 ± 5.1	NS
SpO_2_(%)	81.4 ± 3.9	91.8 ± 1.3	*p* = 0.007^**^
Wheezing	100% (*n* = 7)	100% (*n* = 8)	NS
Severe or serious asthma attack on Japanese guideline^†^	100% (*n* = 7)	62.5% (*n* = 5)	NS
Severe attack on ATS/ERS statement ^††^	100%(*n* = 7)	100%(*n* = 8)	NS
PaCO_2_ ≥ 45 Torr	66.7%(4/6)	0% (0/6)	*p* = 0.014^*^
SpO_2_ ≤ 88%	71.4% (5/7)	22.2% (2/8)	NS
Duration of respiratory failure (days)	5.7 ± 2.5	3.7 ± 1.8	NS
Duration of wheezing (days)	6.7 ± 1.1	7.1 ± 1.8	NS
Duration of steroid treatment (days)	13.7 ± 3.8	12.9 ± 3.5	NS
Duration of hospital stays (days)	7.3 ± 2.0	7.3 ± 1.8	NS

* *p* < 0.05

** *p* < 0.01. All data are presented as (mean ± SD).

**FIGURE 2:**
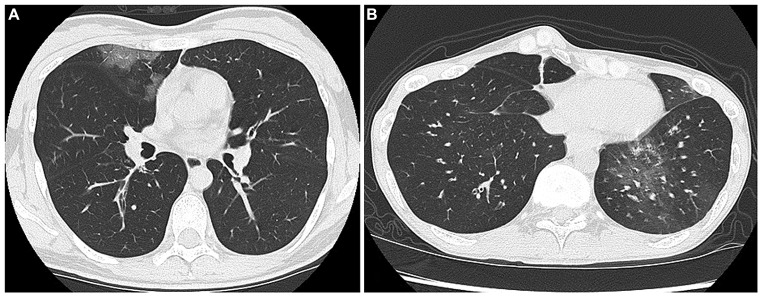
**(A)** Shows a patchy GGO with thickening of interlobular septa in a 28-year-old woman who was admitted during an asthma attack induced by HRV-A. **(B)** Also shows GGO in a 62-year-old man with an asthma attack caused by HRV-C infection.

Interestingly, the incidence of ground glass opacities (GGO) on high resolution computed tomography seemed to be higher for virus-positive hospitalized patients than for virus-negative patients, but it did not reach statistical significance. For example, **Figure 2A** shows a patchy GGO with thickening of interlobular septa in a 28-year-old woman who was admitted during an asthma attack induced by HRV-A. **Figure 2B** also shows GGO in a 62-year-old man with an asthma attack caused by HRV-C infection. These GGO in both patients could only be detected in HRCT, not in chest X-ray.

These results suggested that HRV was the major cause of virus-induced asthma, and was possibly involved in lower airway or lung parenchyma features, appearing as GGO. Viral infection significantly exaggerated the respiratory status (low SpO_2_ and hypercapnea) when compared to that of virus-negative asthma exacerbative patients at the time of admission. Indeed, in recent years, HRV has been recognized as a common cause of hospital admission, both as an agent of bronchopneumonia and through exacerbation of chronic pulmonary conditions, even in the elderly over 65 years of age (Pierangeli et al., 2011).

Curiously, after initiation of treatment with intravenous steroid, both the virus-positive and -negative groups had no significant difference in duration of respiratory failure, wheezing, days in hospital, and even in the time required for steroid treatment.

## TREATMENT

No established treatment for prevention of HRV-induced asthma is available, and we describe the exploratory interventions as follows.

### INHALED CORTICOSTEROID

Inhaled corticosteroid (ICS) is the main drug for regular asthma therapy. ICS treatment improved airway hyperresponsiveness in asthmatic patients experimentally challenged with HRV, however, ICS treatment did not reduce accumulation of inflammatory cells, except for eosinophils in bronchial epithelium (Grunberg et al., 2001). Double-stranded RNA (dsRNA), a viral product and a ligand for the Toll-like receptor-3 (TLR3), upregulates the expression of inflammatory chemokines in airway epithelial cells. Matsukura et al. (2013) reported that treatment of BEAS-2B cells with fluticasone propionate significantly and dose-dependently inhibited dsRNA-induced expression of CCL5, CXCL8, and CXCL10 protein and mRNA. To confirm the effect on ssRNA, such as that of HRV, would need further studies.

### LEUKOTRIENE RECEPTOR ANTAGONIST

Leukotriene receptor antagonist was prescribed in asthmatic patients with or without ICS. Montelukast treatment did not improve asthma control or cold symptom scores when HRV were experimentally inoculated into mild asthmatics, or healthy subjects (Kloepfer et al., 2011). It is uncertain whether leukotriene receptor antagonist treatment is effective in the reduction of asthma symptoms associated with HRV infection.

### ANTI-IgE THERAPY

Zambrano et al. (2003) reported that high serum IgE levels in mildly asthmatic children with experimental HRV infection may be associated with enhanced lower respiratory symptoms and elevation of inflammatory markers, such as nasal eosinophil cationic protein and expired nitric oxide, than those of healthy subjects and/or low IgE asthmatic patients. The prevalence of asthma was closely associated with the serum IgE levels standardized for age and sex (Burrows et al., 1989), and airway hyperresponsiveness appears to be closely linked to the allergic diathesis, as reflected by the serum total IgE level (Sears et al., 1991). Omalizumab, an anti-IgE monoclonal antibody, was indicated in inadequately controlled moderate-to-severe persistent allergic asthma patients who were treated with high dose ICS. Durrani et al. (2012) showed that the IgE receptor FcεRI is inversely associated with IFN-α and IFN-λ1 secretion when plasmacytoid dendritic cells derived from allergic asthmatic children were challenged with HRV. Omalizumab downregulates FcεRI expression on dendritic cells (Prussin et al., 2003), which may reduce exacerbation of asthma associated with increased production of IFNs, through FcεRI.

### ANTI-VIRAL TREATMENT

No drugs are clinically used in HRV infection, although several drugs have been tried for treatment and prevention of HRV infection. These drugs are summarized in a review (Jacobs et al., 2013). IFNs had a potential protective role in viral induced asthma (Cakebread et al., 2011; Gaajetaan et al., 2013). Becker et al. (2013) showed that exogenous IFN-α, IFN-β, IFN-λ1, and IFN-λ2 inhibited HRV replication in BECs from healthy donors.

### MACROLIDE THERAPY

Macrolides are known to possess anti-inflammatory and immunomodulatory actions extending beyond their antibacterial activity in pulmonary inflammatory disorders (Takizawa et al., 1995; Min and Jang, 2012). Erythromycin inhibits HRV infection by reducing ICAM-1 expression on the surface of human tracheal epithelial cells, and modulates inflammation by suppressing the production of proinflammatory cytokines (Suzuki et al., 2002).

### OTHER AGENTS

Yamaya et al. (2014) reported that the mucolytic drug ambroxol hydrochloride, antibiotic drug of levofloxacin (Yamaya et al., 2012b), and bronchodilators (Tiotropium, Tulobuterol, and Procaterol) for asthma or COPD (Yamaya et al., 2011, 2012a, 2013) may have a beneficial effect in HRV infection, by inhibiting HRV replication and partly reducing ICAM-1 expression and acidic endosome production, via the inhibition of NF-kappaB activation (Yamaya, 2012).

## SUMMARY

We reviewed the previous reports regarding HRV-induced asthma exacerbations, together with our results from an institutional prospective study. HRV is a major pathogen for asthma exacerbations, and certainly associated with more serious clinical conditions such as hypoxemia or hypercapnea in hospitalized patients. Further accumulation of evidence of virus-induced asthma for multidisciplinary assessment would be helpful for physicians in recognizing the condition or understanding the pathogenic mechanisms.

## Conflict of Interest Statement

The authors declare that the research was conducted in the absence of any commercial or financial relationships that could be construed as a potential conflict of interest.
